# Green Composite Sensor for Monitoring Hydroxychloroquine in Different Water Matrix

**DOI:** 10.3390/ma14174990

**Published:** 2021-08-31

**Authors:** Danyelle M. de Araújo, Suelya da Silva M. Paiva, João Miller M. Henrique, Carlos A. Martínez-Huitle, Elisama V. Dos Santos

**Affiliations:** 1 Laboratório de Eletroquímica Ambiental e Aplicada, Universidade Federal do Rio Grande do Norte, Lagoa Nova 59072-900, Brazil; danyelle.quimica@yahoo.com.br (D.M.d.A.); suelyapaiva@live.com (S.d.S.M.P.); joaomiller@ufersa.edu.br (J.M.M.H.); 2 National Institute for Alternative Technologies of Detection, Toxicological Evaluation and Removal of Micropollutants and Radioactives (INCT-DATREM), Institute of Chemistry, Universidade Estadual Paulista, P.O. Box 355, Araraquara 14800-900, Brazil

**Keywords:** hydroxychloroquine, COVID-19, cork, graphite, differential pulse voltametric, environmental application

## Abstract

Hydroxychloroquine (HCQ), a derivative of 4-aminoquinolone, is prescribed as an antimalarial prevention drug and to treat diseases such as rheumatoid arthritis, and systemic lupus erythematosus. Recently, Coronavirus (COVID-19) treatment was authorized by national and international medical organizations by chloroquine and hydroxychloroquine in certain hospitalized patients. However, it is considered as an unproven hypothesis for treating COVID-19 which even itself must be investigated. Consequently, the high risk of natural water contamination due to the large production and utilization of HCQ is a key issue to overcome urgently. In fact, in Brazil, the COVID-19 kit (hydroxychloroquine and/or ivermectin) has been indicated as pre-treatment, and consequently, several people have used these drugs, for longer periods, converting them in emerging water pollutants when these are excreted and released to aquatic environments. For this reason, the development of tools for monitoring HCQ concentration in water and the treatment of polluted effluents is needed to minimize its hazardous effects. Then, in this study, an electrochemical measuring device for its environmental application on HCQ control was developed. A raw cork–graphite electrochemical sensor was prepared and a simple differential pulse voltammetric (DPV) method was used for the quantitative determination of HCQ. Results indicated that the electrochemical device exhibited a clear current response, allowing one to quantify the analyte in the 5–65 µM range. The effectiveness of the electrochemical sensor was tested in different water matrices (in synthetic and real) and lower HCQ concentrations were detected. When comparing electrochemical determinations and spectrophotometric measurements, no significant differences were observed (mean accuracy 3.0%), highlighting the potential use of this sensor in different environmental applications.

## 1. Introduction

COVID-19 is caused by a new strain of the coronavirus, which is associated with the same family of viruses as severe acute respiratory syndrome (SARS) and some types of the common cold [1]. As of May 19, 2021, the COVID-19 pandemic has caused more than 3.4 million deaths worldwide [2], and to date, no drug has been proven to target this virus. However, hydroxychloroquine (HCQ) has received remarkable attention as a treatment option for COVID-19 (for example, in the United Sates, Brazil, China, and India) [3]. HCQ is a halogenated aminoquinoline that exhibits wide biological activity and is often used as an antimalarial drug [4]. In Brazil, hydroxychloroquine and ivermectin are included in COVID-19 kits, as a pre-treatment option. However, no scientific results support the effectiveness of these drugs in preventing COVID-19 infection or as a treatment option. Additionally, several people have taken these drugs over a protracted period and the subsequent environmental release of these drugs has resulted in their classification as emerging water pollutants [5,6].

The treatment of wastewater from industrial or municipal sources is undoubtedly a global priority topic for research and development (R&D), primarily because it is on the 2030 agenda for the sustainable development goals (SDG). The presence of a wide variety of pollutants with a wide range of compositions in relevant aquatic environments has been reported, including in rivers and lagoons [7]. Recent researches have demonstrated that HCQ is present in wastewater discharge [8,9]. Additionally, considering its chemical and biological properties, there is a high potential for HCQ persistence and bioaccumulation in vegetation and groundwater, and consequently, it could be associated with soil pollution [6,8,10]. Thus, the HCQ determination is an important monitoring parameter to determine its potential as contaminant [11]. 

HCQ has been previously determined by different analytical methods, some of which are documented in the United States Pharmacopeia and British Pharmacopeia [12,13]. Additionally, chromatographic techniques have been employed, which may have disadvantages such as the need for sample pretreatment, high consumption of chemicals, a long analysis time, and large amounts of waste. Therefore, electrochemical methods have recently received great attention because of their advantages over chromatographic methods, such as a shorter analysis times, lower equipment cost, lower consumption of chemicals, high sensitivity, and the simplicity of preparation [14,15,16,17].

Electroanalytical techniques, such as voltammetry, amperometric potentiometry, etc., which have been developed over the last decades, can provide more accurate and reproducible data. These advantages are based on the fabrication and use of sensors/electrodes [18,19,20]. Major advances in this area are associated with the creation of novel electrodes or electrodes subjected to some type of modification to expand/improve their detection and quantification limits. In addition, the speciation conditions and electrolytes used are fundamental to achieving the desired results. Most recent works have been carried out toward the production carbonaceous-derived electrodes in order to be used in different application such as monitoring compounds and production of hydrogen obtained through green and sustainable materials [21,22]. 

Recently, a low-cost green sensor, which was made from graphite/cork, was proposed for detecting caffeine [17], paracetamol [15], caffeine and paracetamol [23], and Pb ions [24] in different matrices. Cork is a natural material that is obtained from the outer bark of the oak tree, *Quercus suber* L. [25,26] and has a microporous honeycomb-like structure. The life cycle of cork produces three qualities of associate different to the suberose tissue: raw cork; reproduction cork from the second stripping; and reproduction cork from subsequent strips [27]. Usually, the cork is obtained from the bark of a tree and periodically removed without harming the tree; typically, every 9–12 years the cork layer is obtained that reached the minimum required thickness. The cork bark must be about 20–25 years old before its bark associate to raw cork [28].

The chemical and physical properties of cork can be altered by specific pretreatments, such as extraction, alkaline washing, and thermal acid treatment [29]. Based on its effectiveness as an electrochemical sensor when it is combined with graphite, herein, we propose a cork–graphite composite electrode for detecting HCQ in water matrices. Firstly, the efficacy of the cork–graphite electrode for quantify HCQ was evaluated in different supporting electrolytes (0.1 M NaCl, 0.1 M NaOH, 0.1 M CH_3_COOH, 0.1 M HCl, 0.1 M Na_2_SO_4_, 0.1 M CH_3_COONa, and 0.1 M H_2_SO_4_) by using differential pulse voltammetry (DPV). Secondly, HCQ detection in real water matrices (river, lagoon, tap water, and ground water) was also achieved. Finally, the selectivity, repeatability, and reproducibility of the cork-graphite sensor were verified.

## 2. Materials and Methods

### 2.1. Reagents and Materials

HCQ sulfate (purity 99%) and graphite powder were purchased from Sigma-Aldrich (São Paulo, Brazil). NaCl, NaOH, CH_3_COOH, HCl, Na_2_SO_4_, CH_3_COONa, and H_2_SO_4_ were purchased from Merck (São Paulo, Brazil). All solutions were prepared using ultra-purified water obtained from a Milli-Q system (Millipore, Natal, Brazil). The raw cork (RAC) that was used in the experimental studies was provided by Corticeira Amorim S.G.P.S., S.A. (Porto, Portugal). The RAC granules were washed twice with distilled water for 2 h at 60 °C to remove impurities and other water extractable components that could interfere with the electrochemical analysis. Before use, the RAC was dried at 60 °C in an oven for 24 h [16]. The HCQ standard solutions were prepared daily using ultra-purified water to prevent photodegradation, to which HCQ is susceptible [13]. 

### 2.2. Preparation of Cork-Graphite Sensor

According our previous work [16], SEM micrographs of raw cork showed a honeycomb structure, which is associated with the macropores. After the pre-washing approach of cork, there are small internal impurities. Based on the results obtained by FTIR, the presence of –OH and –CH_3_ signals, at 3440–3400 and 2920–2850 cm^−1^, and the disappearance of the C=O stretch bands at 1745–1715 cm^−1^ (characteristic of ester groups, originating mainly from suberin), were the most important modifications of the material, which could give significant insights in terms of the cork surface to interact with the contaminants dissolved in water, as already reported by other authors.

### 2.3. Electrochemical Measurements

Electrochemical analyses were performed on an Autolab PGSTAT302N (Metrohm, Zurich, Switzerland) that was controlled with GPES software (4.0) and consisted of a three-electrode cell, using Ag/AgCl (3.0 M KCl), Pt wire and a cork–graphite sensor (GrRAC, (geometrical area of approximately 0.45 mm^2^)) as the reference, auxiliary, and working electrodes. The oxidation of HCQ was investigated using cyclic voltammetry (CV), which was performed with different supporting electrolytes (0.1 M NaCl, 0.1 M NaOH, 0.1 M CH_3_COOH, 0.1 M HCl, 0.1 M Na_2_SO_4_, 0.1 M CH_3_COONa, and 0.1 M H_2_SO_4_). The effect of the potential scan rate (*v*) on the HCQ response was evaluated by varying this parameter in the range of 10 to 500 mV s^−1^ for different CV analyses. The DPV parameters were as follows—modulation time: (≥0.002 s), 0.05 s; interval time: (≥0.10 s), 0.5 s); initial potential: 1.0 V; final potential: 1.7 V; step potential: 0.00495 V; modulation amplitude: 0.01995 V; potential scan rate: 100 mV s^−1^; and agitation time: 30 s. The optimized parameters were used for all measurements. All analyses were performed in triplicate. All electrochemical analyses were conducted without deaeration, at room temperature (25 ± 2 °C). For comparative purposes, HCQ determination was also carried out spectrophotometrically using a UV-vis spectrophotometer (Bel Photonics, SP 2000 UV, Berlin, Germany) set at 342 nm, with a quartz cuvette and 0.01 mol L^−1^ HCl solution as the solvent [13]. For the determination of HCQ in different water matrices (river, lagoon, tap water, and ground water), the water samples were spiked with a known quantity of a standard solution of HCQ. The final HCQ concentration was determined using the standard addition method. For this purpose, the samples were doped with different concentrations of HCQ (5–69 µM). Thereafter, 10 mL aliquots of each sample were diluted in the supporting electrolyte solution, and the final solutions were analyzed.

## 3. Results

### 3.1. Effects of Supporting Electrolyte

**Figure 1:**
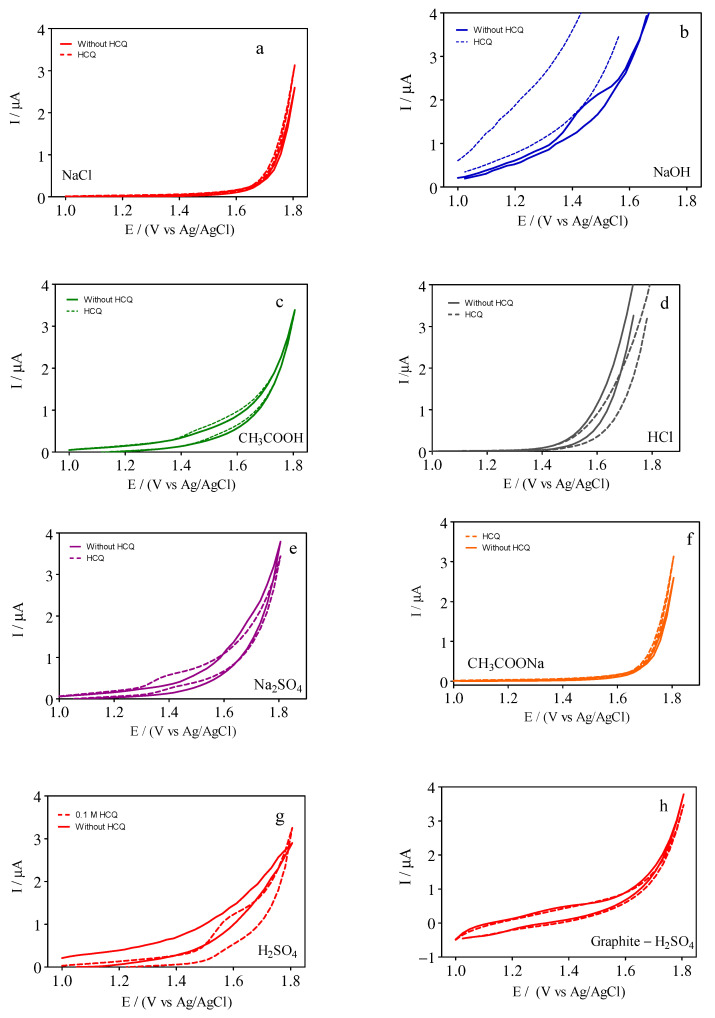
CV curves recorded in absence (full lines) and in presence of 10 µM HCQ (dashed lines) for different supporting electrolytes: (**a**) 0.1 M NaCl, (**b**) 0.1 M NaOH, (**c**) 0.1 M CH_3_COOH, (**d**) 0.1 M HCl, (**e**) 0.1 M Na_2_SO_4_, (**f**) 0.1 M CH_3_COONa, and (**g**) 0.1 M H_2_SO_4_. (**h**) CV responses of graphite electrodes in 0.1 M H_2_SO_4_. All experimental conditions have been reported in Section 2.2.

No electrochemical work corresponding to HCQ has been reported previously in the literature using a GrRAC sensor. Then, the supporting electrolyte effect was investigated based on the CV responses of 10 µM HCQ with a GrRAC sensor in 0.1 M NaCl, 0.1 M NaOH, 0.1 M CH_3_COOH, 0.1 M HCl, 0.1 M Na_2_SO_4_, 0.1 M CH_3_COONa, and 0.1 M H_2_SO_4_, as shown in Figure 1. In terms of the most favorable conditions, the GrRAC sensor in the presence of 0.1 M H_2_SO_4_ showed the most well-defined anodic peak corresponding to HCQ. As can be observed in Figure 1, HCQ exhibited one well-defined oxidation peak in the presence of 0.1 M NaOH (Figure 1b), 0.1 M Na_2_SO_4_ (Figure 1e) and 0.1 M H_2_SO_4_ (+1.3 V and +1.6 V, (Figure 1g)), respectively. This result shows that HCQ is more easily oxidized in the presence of 0.1 M NaOH (Figure 1b) and 0.1 M Na_2_SO_4_ (Figure 1e) when compared to that of 0.1 M H_2_SO_4_ (Figure 1g). However, lower background currents were mainly observed when 0.1 M Na_2_SO_4_ (Figure 1e) and 0.1 M H_2_SO_4_ (Figure 1g) were used as supporting electrolytes in the presence of HCQ. Meanwhile, no significant signals were observed when 0.1 M NaCl (Figure 1a), 0.1 M CH_3_COOH (Figure 1c), 0.1 M HCl (Figure 1d), 0.1 M CH_3_COONa (Figure 1f) were used as supporting electrolytes. Additionally, no reduction peak was observed, in all cases (see, Figure 1), in the reverse scan, confirming the irreversibility of the electro-oxidation process of HCQ in all supporting electrolytes.

It is important to remark that, when real water matrices will be analyzed, then, a preconditioning acidic strategy is used to preserve the water properties avoiding a quick biological degradation before the use of electrochemical determinations [30]. In this frame, 0.1 M H_2_SO_4_ was selected as a supporting electrolyte in subsequent experiments, as already reported by Deroco et al. [31]. A comparison between GrRAC and graphite sensors was also carried out under acidic pH conditions (0.1 M H_2_SO_4_) in order to verify the improvements on the HCQ signal when RAC was used as modifier. As can be seen in Figure 1, an important enhancement in voltammetric response was achieved, in the presence of 10 µM of HCQ, when GrRAC (Figure 1g) was applied as electrochemical sensor in respect of a non-RAC modified electrode (Figure 1h). This result clearly evidences the potential utilization of GrRAC as HCQ monitoring device as well as demonstrating undoubtedly the advances on the current sensibility when RAC is mixed with graphite. In order to understand the different behaviors registered by CV analysis, the peak current of the GrRAC sensor, under diverse experimental conditions, was considered and estimated to the according electroactive surface area (A_real_) of 0.12 mm^2^, see Table 1. It can be observed that the GrRAC sensor in the presence of H_2_SO_4_ contributes most effectively for the oxidation of HCQ. 

### 3.2. Effects of Scan Rate

**Figure 2:**
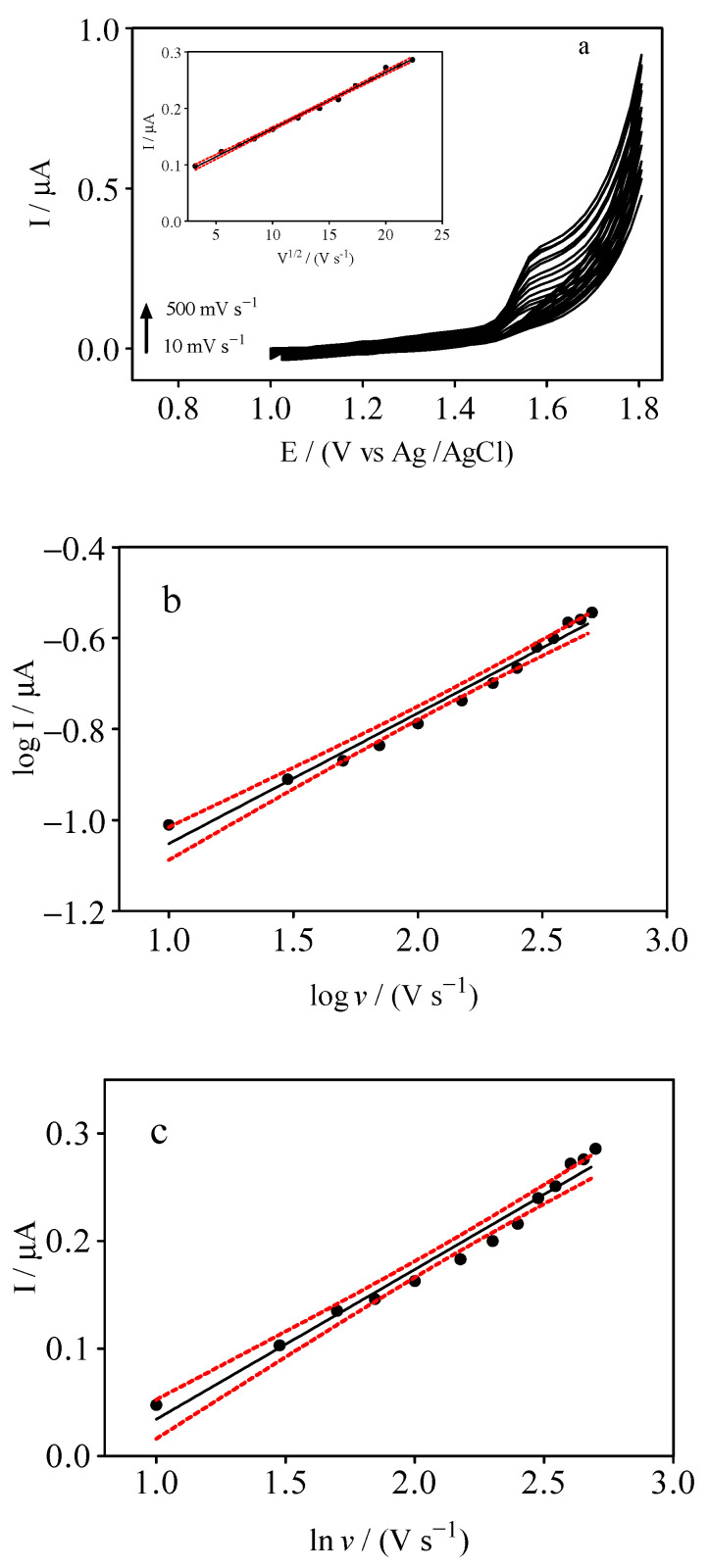
(**a**) Cyclic voltammograms registered at different scan rates (10–500 mV s^−1^) by using a cork–graphite (GrRAC) sensor in the presence of 10 mM HCQ in solution 0.1 M H_2_SO_4_. Inset graphic: Anodic peak currents versus square root of the scan rate, (**b**) Scan rate (log *I* vs. log *v*) in the presence of HCQ, and (**c**) The linear relation between Ep and ln υ obtained at different scan rates.

**Figure 3:**
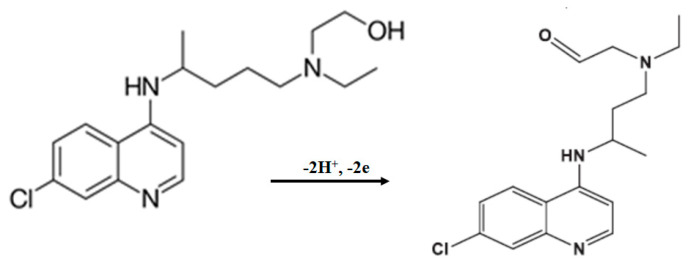
Proposed electrochemical oxidation mechanism of HCQ for cork-graphite (GrRAC) in 0.1 M H_2_SO_4_ solution, in agreement with the existing literature [42,43].

As described in the previous section, the supporting electrolyte influenced the redox processes at the electrode surface. The effect of the scan rate (10–500 mV s^−1^) on the electrochemical HCQ response was also investigated using 10 µM HCQ in 0.1 M H_2_SO_4_ solution. As shown in Figure 2a, increasing the scan rate, an increase on the anodic peak current (*I_pa_*) was observed; however, the oxidation potential was also slightly shifted to more positive potential values. The peak current was determined using GPES software (version 4.0) by extrapolating from the baseline of the peak current measurement [32]. The relationship between the peak current and the square root of the scan rate (*I* vs. *v*^1/2^), as well as the relationship between the logarithm of the peak current and the logarithm of the scan rate (log *I* vs. log *v*), both allowed us to understand the mass transport behaviors, Figure 2b. The linear relationship between the oxidation peak current and the square root of the scan rate ((*I* vs. *v*^1/2^), see inset Figure 2a) confirmed that HCQ oxidation is a diffusion-controlled process. The relationship between *I_pa_* (µA) and log *v* (mV s^−1^) can be expressed by the following equations: *I_pa_* (µA) = 0.28 log *v* (mV s^−1^)−1.3 (*R*^2^ = 0.979), Figure 2c. The slope estimated was about 0.5, confirming that HCQ oxidation was mainly controlled by the diffusion process, as previously observed with other organic compounds for GrRAC sensors [33,34]. Additionally, the absence of significant nonlinearity was also visually verified, as recommended by IUPAC [35,36] and the literature [37]. The number of electrons (*n*) is an important parameter for evaluating a completely irreversible process. *E_pa_* versus ln *v* was plotted in order to determine *n* involved in the oxidation mechanism of HCQ [38]. Figure 2c shows that *E_pa_* was shifted towards positive potentials with an increase in the scan rate. Meanwhile, the linear dependence between *E_pa_* and ln *v* can be expressed by the following equation: *E_pa_*/V = 0.14 − 0.10 ln *v*. The slope of the plot of *E_pa_* versus ln *v* (assuming *α* = 0.5) can be used to calculate *n*, using the Laviron theory [38,39].

(1) $$E_{P}\left(V\right)=E^{0^{\prime }}-\frac{RT}{\alpha nF}ln\frac{RTk_{s}}{\left(1\right)\alpha nF}+\frac{RT}{\alpha nF}lnv$$

where *E°* is the formal redox potential; K_s_ is the standard heterogeneous reaction rate constant; *α* is the electron transfer coefficient that assumes a value of 0.5, owing to the irreversible electrochemical behavior in acidic media; *n* is the number of electrons transferred in the system, and R, *T*, and F have their usual meanings (R = 8.314 J mol^−1^ K^−1^, *T* = 298 K, F = 96,485 C mol^−1^). *E°* = 0.12 V (formal standard potential) was estimated from the linear relationship between *E_pa_* and ln *v* by extrapolating from *v* = 0. From this calculation, it was found that two electrons were involved in the HCQ oxidation under acidic pH conditions (Figure 3). In fact, the value of *E°* confirms the results reported by Mahnashi et al. [40], Decoro et al. [31], Khoobi et al. [41], and Arguelho et al. [11] which have suggested that the electrooxidation of HCQ involved the nitrogen atom of the alkylamino side chain and the N-heterocyclic nitrogen of the aminoquinoline moiety of hydroxychloroquine, respectively (Figure 3). *E°* was obtained from the slope of *E_pa_* versus ln *v* (assuming *α* = 0.5). Consequently, the heterogeneous electron transfer rate constant (K_s_ = 6.5 × 10^5^ s^−1^) could be estimated using Equation (1). 

### 3.3. DPV Analytical Curve

**Figure 4:**
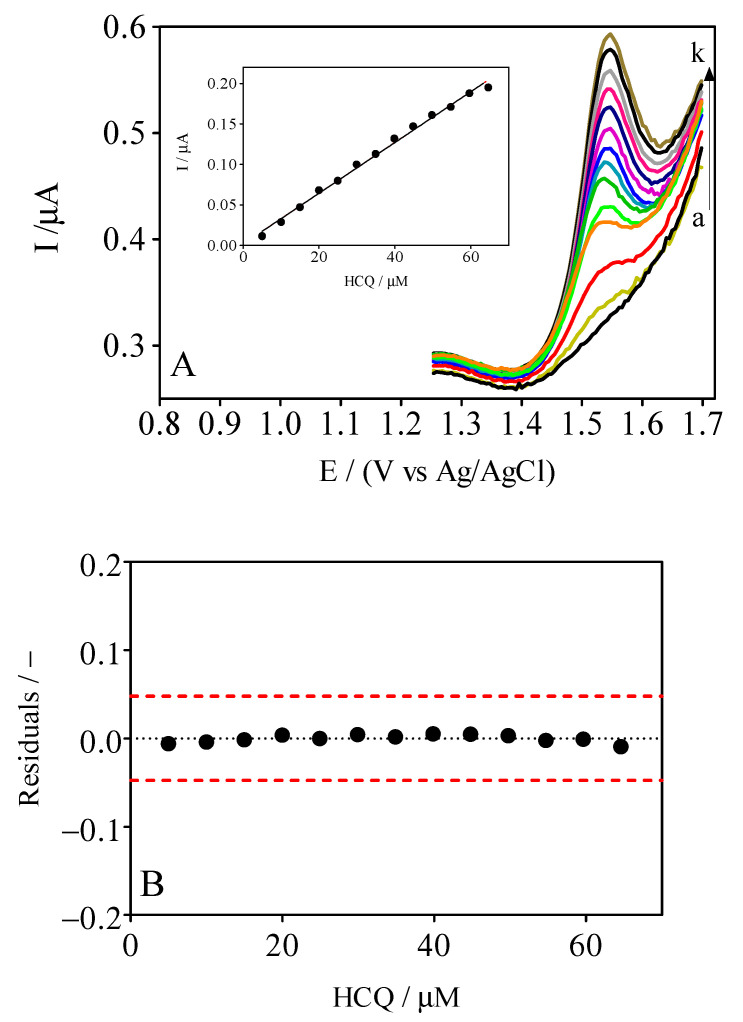
(**A**) DPV curves for electrochemical cork–graphite sensor in 0.1 M H_2_SO_4_ by adding standard HCQ solution (10 mM) in well-known concentrations: (a) 5,0 (b) 25, (c) 10, (d) 15, (e) 20, (f) 25, (g) 30, (h) 35, (i) 40, (j) 45, and (k) 65 µM. Inset: Plot of electrochemical responses, in terms of current, as a function of HCQ concentration. (**B**) Graphic representation of the residuals behavior, which confirms the linearity of the calibration curve.

A protocol for the electrochemical determination of HCQ was developed using the DPV approach with a GrRAC electrode, and it was optimized using different electrolytes. However, based on the CV responses, H_2_SO_4_ was employed as the main supporting electrolyte. Figure 4 shows the DPV data for HCQ in H_2_SO_4_; the resulting high-intensity curve is shown in the inset of Figure 4. As previously observed for the oxidation of HCQ in the CV analysis, in terms of the voltammetric current response, acidic H_2_SO_4_ conditions produced the best oxidation electroactivity signal, and it was more intense and higher at GrRAC sensor than that obtained in the graphite electrode. This behavior is associated with the morphology of the GrRAC electrode, which promoted chemical and electrochemical interactions, associated with the composition of the cork [16]. The analytical curve obtained for the GrRAC electrochemical sensor is represented and it can be observed that the peak current (Ip) increased linearly with the concentration of HCQ in the range from µM, and the linear regression equation (Ip vs. C) was obtained, Ipa (µA) = 3.0 × 10^−3^ × [HCQ] − 1.6 × 10^−3^ (R^2^ = 0.994). The analytical curve was obtained by considering the peak intensity as a function of the HQC concentration and evaluating a range between 5–65 µM analyte concentrations. The residuals of the regression curve were also included in Figure 4 in order to confirm visually the absence of significant non-linearity, as recommended by IUPAC [35,36] and the literature [37]. The limit of detection (LOD) for the GrRAC sensor was estimated according this equation: 3.3 × S_y/x_/b, where S_y/x_ is the residual standard deviation and b is the slope of the calibration plot [36]. The LOD and LQ were found to be 1.05 and 3.15 µM. Another parameter that was evaluated in this work consisted to the stability of the sensor used that shows good performance for at least two months of intensive use as well as reproducibility when four analytical curves were achieved by using the same concentration range and sensor, obtaining no significant variations. 

There are few studies in the literature reporting on the quantification of HCQ. Table 2 shows these works, providing a comparison of the main analytical parameters for quantification HCQ, such as electrode composition, supporting electrolyte, method and LOD. In addition, the analytical performance such as linear range and LOD were compared with the previously reported sensor for HQC quantification. As can be confirmed, the proposed sensor has a good electroactivity performance in comparison with the methods reported previously. Ghoreishi et al. [43] reported the use of glassy carbon modified with N,N′-bis[(E)-(1-pyridyl) methylidene]-1,3-propanediamine (PMPD) self-assembled monolayer (SAM) for detection of HCQ achieving a LOD of 25.8 µM. In another study, glassy carbon was modified with N,N-bis[(E)-(1-pyridyl) methylidene]-1,3-propanediamine (PMPDA) self-assembled monolayer (SAM) with which was assessed a LOD of 0.0046 µM in the presence of acetaminophen [44]. Thus, based on the results in the existing literature, the main advantages of the developed sensor are the use of a 100% natural and sustainable material as the modifier, affording low LOD values for HCQ detection. This novel sensor material seems to offer a fast, reliable, economic, and simple way for quantifying HCQ. The concentration capability of the cork modification, due to its affinity towards the analyte, produces a significant gain of sensitivity which can be used to identify and quantify HCQ in different samples without consuming time or aggressive reagents pre-treatment.

### 3.4. Determination of HCQ in Water Samples

**Figure 5:**
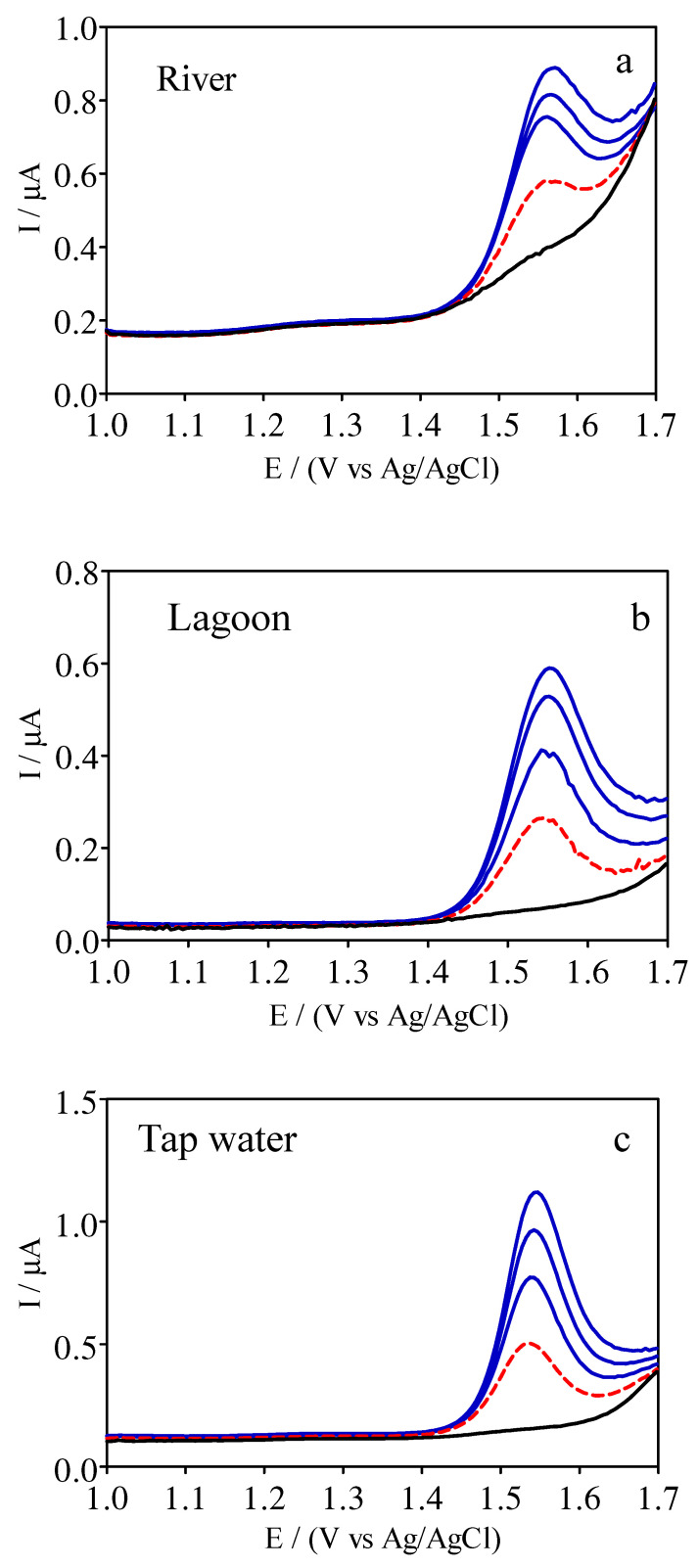
Results obtained for determining HCQ in different water matrices ((**a**) river, (**b**) lagoon, and (**c**) tap water) by the standard additions method using the DPV approach with a GrRAC sensor ((**—**) standard additions of 10 mM HCQ in 0.1 M H_2_SO_4_ (200, 400 and 600 µL) as well as (**—**) supporting electrolyte and (**- - -**) water sample).

To investigate the electrochemical sensor suitability for environmental monitoring, the new, low-cost, green device was used to determine the HCQ concentration in water samples from river, lagoon, and tap water sources. Table 3 shows the concentration of HCQ that was added to all samples assayed using the proposed sensor and the reference spectrophotometric method. Thus, acceptable recovery values were obtained for all types of samples, which ranged from 120 ± 0.52% for river samples, 111 ± 0.8% for lagoon samples, and 121 ± 0.35% for tap water samples (Table 3). The HCQ signal was confirmed by intensification of the peaks associated with the addition of different volumes of standard HCQ solution to the samples (Figure 5, (a) river, (b) lagoon, and (c) tap waters). It is important to highlight that the results were obtained with acceptable standard deviations and confidence intervals, within 95%. This information can be used to identify false positives and false negatives (*α* = β = 0.05), as already indicated by experts in the field [36]. Additionally, the experimental recoveries were close to 100%, although the electrochemical signal was validated in different matrices. Based on the data obtained, the proposed sensor can be used to determine the HCQ concentration in environmental samples. 

On the other hand, a river water sample presented a positive result to HCQ detection after spectrophotometric evaluation, registering a HCQ concentration of approximately (3.27 ± 0.04) × 10^−3^ mM. After that, the same river water sample was evaluated by the DPV approach with a GrRAC sensor, achieving (2.85 ± 0.06) × 10^−3^ mM. This figure evidenced the HCQ water pollution as well as the potential use of cork as a modifier of graphite for monitoring water quality, even when the difference between both measurements is about 12%, which can be considered acceptable.

It is important to highlight that the water and wastewater samples contain numerous inorganic and organic compounds present, which can interfere in the functioning of the electrochemical sensor, along with the target analyte. For this reason, the standard addition method is recommended for diminishing the matrix effect on the current-response sensibility. However, an additional study was carried out with a lagoon water sample (which was previously acidified to avoid sample decomposition) where the electrochemical sensor’s response, in terms of current, was verified. As can be observed in Figure S1 in the Supplementary Material (SM), a good performance of the proposed electrochemical sensor was achieved, showing a lower matrix effect during analytical curve construction as well as good linearity in the current responses as a function of concentration (see inset in Figure S1). The LOD was found to be 2.71 µM, which is only 2.58-folds higher than the LOD obtained in this work by using distilled water (see Table 2). Then, the determination of HCQ in water matrices was adequately attained by the standard addition method, as is reported in Figure 5. 

## 4. Conclusions

This study demonstrates that cork–graphite-based sensors provide a fast, reliable, cost-effective, and simple method for quantifying HCQ concentrations in different water samples. The sensor exhibited the best affinity and a higher sensitivity for HCQ when 0.1 M H_2_SO_4_ was used as the electrolyte support. Based on the results, the sensor exhibited a good response for HCQ determination, despite the matrix effects in dilute solutions. The affinity of the cork for the analyte substantially improved the sensitivity for the evaluated analytes. Furthermore, the proposed approach is precise, with an LOD of 1.05 μM. Compared to other analytical methods, this approach is reproducible and less expensive, both in terms of time and materials. 

The composite electrode could be also effectively used for the determination of HCQ in other media. However, as it pertains to the ideal physical and chemical properties that favor interactions with the analytes, more experiments are necessary to better understand the chemical/electrochemical processes that take place on the cork surface, the cork-absorbent interactions when a current is applied, or when the cork participates as a mediator. Finally, even if the LOD reported in this work (1.05 µM) is slightly above the detection limit established by other materials (see Table 2), there is room for improvements; for example, the size of the electrochemical sensor which could be reduced, the use of other carbon-based modifiers [21,22] which could enhance its selectivity and sensitivity, and consequently, improving the LOD. Finally, integrated environmentally-friendly electrochemical technologies [15] could be proposed for removing and detecting pollutants by using sensors and advanced oxidation/reduction processes [15,47,48] because it is possible to design small portable devices for monitoring pollutants before and after their elimination from water which benefit the use of specific strategies in real time. In fact, regarding the treatment of the river water sample in which was detected a HCQ contamination, reported in this work, it could be treated by electrochemical oxidation technology [15,48,49] and monitored by using the cork–graphite-based sensor proposed here. 

## Figures and Tables

**Table 1:** E_p_ and peak-current of the GrRAC sensor for different electrolytes in the presence of HQC in 0.1 M H_2_SO_4_ at 100 mV s^−1^.

Electrolyte	E_p_/mV	i_p_/µA	i_p_/µA mm^−2^
NaCl	-	-	-
NaOH	+1.45	1.89	15.7
CH_3_COOH	+1.45	0.33	0.28
HCl	-	-	-
Na_2_SO_4_	+1.40	0.058	0.48
CH_3_COONa	-	-	
H_2_SO_4_	+1.55	1.11	9.25

**Table 2:** HCQ determinations by using different sensors, experimental conditions, and electroanalytical techniques. Comparison with the result obtained in this work.

Electrodes	Method	Electrolyte	Linear Range µM	LOD/µM	Ref.
GrRAC	DPV	0.1 M H_2_SO_4_	5–65	1.05	This work
^5^ Modified carbon paste	Potentiometric titration	0.01 M Sodium tetraphenylborate	1–1000	0.78 0.46	[45]
Glassy carbon	DPV	B-R buffer (pH = 4.0)	35–100	0.336	[11]
^4^ VS2 QDs	DPV	B.R. buffer (pH = 6.0)	0.84–22.5	0.277	[40]
^6^ BDD	SWV	0.1 M H_2_SO_4_	0.1–1.9	0.06	[31]
^3^ MWCNTs/CPE	AdSDPV	Phosphate buffer (pH = 8.0)	0.57–100	0.006	[42]
^1^ GC-PMPD SAM	DPV	B–R buffer (pH = 8.0)	0.05–12.8 12.3–111	0.00451	[43]
^1^ GC-PMPDA SAM	DPV	B-R buffer (pH = 8.0)	0.09–10.2 10.2–98.2	0.00465	[44]
Schiff’s base modified GCE	DPV	B.R. solution (pH = 6.0)	0.007–11.9	0.00465	[41]
^2^ β-CD-AuNP	DPV	Phosphate buffer (pH = 6.0)	0.01–0.05	0.00261	[46]

^1^ N,N0-bis[(E)-(1-pyridyl) methylidene]-1,3-propanediamine (PMPD) self-assembled monolayer (SAM); ^2^ Gold electrodes modified with β-CD-AuNP; ^3^ Multi-walled carbon nanotubes (MWCNTs) modified carbon paste electrode; ^4^ Vanadium disulfide quantum dots; ^5^ Carbon paste sensors based on hydroxychloroquine-phosphotungstate (HCQ-PTA) ion-pair or β-cyclodextrin (β-CD) ionophore and dibutyl phthalate (DBP) as plasticizer; ^6^ Boron-doped diamond.

**Table 3:** HCQ concentration in water samples from river, lagoon, and tap water sources. Well-known HCQ concentration was added (standard additions of 10 mM HCQ in 0.1 M H_2_SO_4_ (200, 400, and 600 µL)) to all samples assayed which were evaluated using the proposed sensor and the reference spectrophotometric method, and reporting the recovery results comparing the results with the spectrophotometric method.

Sample	Present Method	HCQ Added/µM	HCQ Found/µM	Recovery (%)
River	UV-vis	69.5	83.67 ± 0.52	120.4
	DPV *	69.5	108.0 ± 8.38	144.0
Lagoon	UV-vis	69.5	84.67 ± 1.18	121.8
	DPV *	69.5	76.20 ± 1.08	111.0
Tap water	UV-vis	69.5	80.00 ± 0.67	110.0
	DPV *	69.5	83.50 ± 0.35	120.0

* Standard additions method using DPV approach with GrRAC sensor.

## Data Availability

Data sharing not applicable.

## Supplementary Materials

The following are available online at www.mdpi.com/article/10.3390/ma14174990/s1, Figure S1: DPV curves for electrochemical cork-graphite sensor in acidified sample of lagoon by adding standard HCQ solution (10 mM) in well-known volumes to obtain: (a) 5.0 (b) 10, (c) 15, (d) 20, (e) 25, (f) 30, (g) 35, (h) 40, (i) 45, (j) 50, (k) 55, (l) 65, (m) 70 µM. Inset: Plot of electrochemical responses, in terms of current, as a function of HCQ concentration. Equation: Ipa (µA) = 3.13 × 10^−3^ × [HCQ] − 5.53 × 10^−4^ (R2 = 0.993).

## References

[1] WHO, IFRC, Unicef (2020). Key Messages and Actions for Prevention and Control in Schools. Key Messag. Actions COVID-19 Prev. Control Sch..

[2] (2021). WHO Coronavirus (COVID-19) Dashboard.

[3] Dagens A., Sigfrid L., Cai E., Lipworth S., Cheung V., Harris E., Bannister P., Rigby I., Horby P. (2020). Scope, quality, and inclusivity of clinical guidelines produced early in the covid-19 pandemic: Rapid review. BMJ.

[4] Fox R. (1996). Anti-malarial drugs: Possible mechanisms of action in autoimmune disease and prospects for drug development. Lupus.

[5] Mooney D., Richards K.G., Danaher M., Grant J., Gill L., Mellander P.E., Coxon C.E. (2021). An analysis of the spatio-temporal occurrence of anthelmintic veterinary drug residues in groundwater. Sci. Total Environ..

[6] Howard P.H., Muir D.C.G. (2013). Identifying new persistent and bioaccumulative organics among chemicals in commerce. III: Byproducts, impurities, and transformation products. Environ. Sci. Technol..

[7] Guo Z., Boeing W.J., Borgomeo E., Xu Y., Weng Y. (2021). Linking reservoir ecosystems research to the sustainable development goals. Sci. Total Environ..

[8] Dabić D., Babić S., Škorić I. (2019). The role of photodegradation in the environmental fate of hydroxychloroquine. Chemosphere.

[9] Bensalah N., Midassi S., Ahmad M.I., Bedoui A. (2020). Degradation of hydroxychloroquine by electrochemical advanced oxidation processes. Chem. Eng. J..

[10] Chen Y.S., Yu S., Hong Y.W., Lin Q.Y., Li H.B. (2013). Pharmaceutical residues in tidal surface sediments of three rivers in southeastern China at detectable and measurable levels. Environ. Sci. Pollut. Res..

[11] Arguelho M.L.P.M., Andrade J.F., Stradiotto N.R. (2003). Electrochemical study of hydroxychloroquine and its determination in plaquenil by differential pulse voltammetry. J. Pharm. Biomed. Anal..

[12] (2007). United States Pharmacopoeia.

[13] (2008). British Pharmacopoeia.

[14] Deroco P.B., Fatibello-Filho O., Arduini F., Moscone D. (2019). Effect of Different Carbon Blacks on the Simultaneous Electroanalysis of Drugs as Water Contaminants Based on Screen-printed Sensors. Electro Anal..

[15] Henrique J.M.M., Monteiro M.K.S., Cardozo J.C., Martínez-Huitle C.A., da Silva D.R., dos Santos E.V. (2020). Integrated-electrochemical approaches powered by photovoltaic energy for detecting and treating paracetamol in water. J. Electroanal. Chem..

[16] Monteiro M.K.S., Da Silva D.R., Quiroz M.A., Vilar V.J.P., Martínez-Huitle C.A., Dos Santos E.V. (2021). Applicability of cork as novel modifiers to develop electrochemical sensor for caffeine determination. Materials.

[17] Monteiro M.K.S., Paiva S.S.M., da Silva D.R., Vilar V.J.P., Martínez-Huitle C.A., dos Santos E.V. (2019). Novel cork-graphite electrochemical sensor for voltammetric determination of caffeine. J. Electroanal. Chem..

[18] Lourenção B.C., Medeiros R.A., Rocha-Filho R.C., Mazo L.H., Fatibello-Filho O. (2009). Simultaneous voltammetric determination of paracetamol and caffeine in pharmaceutical formulations using a boron-doped diamond electrode. Talanta.

[19] Katseli V., Economou A., Kokkinos C. (2020). A novel all-3D-printed cell-on-a-chip device as a useful electroanalytical tool: Application to the simultaneous voltammetric determination of caffeine and paracetamol. Talanta.

[20] Silva K.N.O., Paiva S.S.M., Souza F.L., Silva D.R., Martínez-Huitle C.A., Santos E.V. (2018). Applicability of electrochemical technologies for removing and monitoring Pb2+ from soil and water. J. Electroanal. Chem..

[21] Zuliani A., Cano M., Calsolaro F., Puente Santiago A.R., Giner-Casares J.J., Rodríguez-Castellón E., Berlier G., Cravotto G., Martina K., Luque R. (2021). Improving the electrocatalytic performance of sustainable Co/carbon materials for the oxygen evolution reaction by ultrasound and microwave assisted synthesis. Sustain. Energy Fuels.

[22] Cova C.M., Zuliani A., Puente Santiago A.R., Caballero A., Muñoz-Batista M.J., Luque R. (2018). Microwave-assisted preparation of Ag/Ag2S carbon hybrid structures from pig bristles as efficient HER catalysts. J. Mater. Chem. A.

[23] Monteiro M.K.S., Santos E.C.M.M., Silva D.R., Martínez-Huitle C.A., dos Santos E.V. (2020). Simultaneous determination of paracetamol and caffeine in pharmaceutical formulations and synthetic urine using cork-modified graphite electrodes. J. Solid State Electrochem.

[24] Silva I.B., de Araújo D.M., Vocciante M., Ferro S., Martínez-Huitle C.A., Dos Santos E.V. (2021). Electrochemical determination of lead using a composite sensor obtained from low-cost green materials: Graphite/cork. Appl. Sci..

[25] Gil L. (2015). New cork-based materials and applications. Materials.

[26] Pintor A.M.A., Ferreira C.I.A., Pereira J.C., Correia P., Silva S.P., Vilar V.J.P., Botelho C.M.S., Boaventura R.A.R. (2012). Use of cork powder and granules for the adsorption of pollutants: A review. Water Res..

[27] Silva S.P., Sabino M.A., Fernandas E.M., Correlo V.M., Boesel L.F., Reis R.L. (2005). Cork: Properties, capabilities and applications. Int. Mater. Rev..

[28] Duarte A.P., Bordado J.C. (2015). Cork—a renewable raw material: Forecast of industrial potential and development priorities. Front. Mater..

[29] Souza R.S., Porto P.S.S., Pintor A.M.A., Ruphuy G., Costa M.F., Boaventura R.A.R., Vilar V.J.P. (2016). New insights on the removal of mineral oil from oil-in-water emulsions using cork by-products: Effect of salt and surfactants content. Chem. Eng. J..

[30] Liwka-Kaszyńska M.S., Kot-Wasik A., Namieśnik J. (2003). Preservation and Storage of Water Samples. Crit. Rev. Environ. Sci. Technol..

[31] Deroco P.B., Vicentini F.C., Oliveira G.G., Rocha-Filho R.C., Fatibello-Filho O. (2014). Square-wave voltammetric determination of hydroxychloroquine in pharmaceutical and synthetic urine samples using a cathodically pretreated boron-doped diamond electrode. J. Electroanal. Chem..

[32] Jakubowska M. (2011). Signal Processing in Electrochemistry. Electroanalysis.

[33] Santos A.M., Wong A., Fatibello-Filho O. (2018). Simultaneous determination of salbutamol and propranolol in biological fluid samples using an electrochemical sensor based on functionalized-graphene, ionic liquid and silver nanoparticles. J. Electroanal. Chem..

[34] Maier S.A. (2007). Plasmonics: Fundamentals and Applications.

[35] Currie L.A. (1995). Nomenclature in Evaluation of Analytical Methods Including Detection and Quantification Capabilities. Pure Appl. Chem..

[36] Danzer K., Currie L.A. (1998). Guideline for calibration in analytical chemistry—Part 1. Fundamentals and single component calibration. Pure Appl. Chem..

[37] Desimoni E., Brunetti B. (2009). About estimating the limit of detection of heteroscedastic analytical systems. Anal. Chim. Acta..

[38] Bard A.J., Faulkner L.R., Bard A.J., Faulkner L.R. (2001). ELECTROCHEMICAL METHODS Fundamentals and Applications.

[39] Laviron E. (1979). General expression of the linear potential sweep voltammogram in the case of diffusionless electrochemical systems. J. Electroanal. Chem..

[40] Mater Mahnashi H., Mahmoud A.M., Saad Alkahtani A., El-Wekil M.M. (2021). Simultaneous electrochemical detection of azithromycin and hydroxychloroquine based on VS2 QDs embedded N, S @graphene aerogel/cCNTs 3D nanostructure. Microchem. J..

[41] Khoobi A., Mehdi Ghoreishi S., Behpour M. (2014). Sensitive and selective determination of hydroxychloroquine in the presence of uric acid using a new nanostructure self-assembled monolayer modified electrode: Optimization by multivariate data analysis. Analyst.

[42] Ghoreishi S.M., Attaran A.M., Amin A.M., Khoobi A. (2015). Multiwall carbon nanotube-modified electrode as a nanosensor for electrochemical studies and stripping voltammetric determination of an antimalarial drug. RSC Adv..

[43] Ghoreishi S.M., Behpour M., Khoobi A., Salavati-Niasari M. (2013). Electrochemical study of a self-assembled monolayer of N,N′-bis[(E)-(1-pyridyl) methylidene]-1,3-propanediamine formed on glassy carbon electrode: Preparation, characterization and application. Anal. Methods.

[44] Khoobi A., Ghoreishi S.M., Behpour M., Shaterian M., Salavati-Niasari M. (2014). Design and evaluation of a highly sensitive nanostructure-based surface modification of glassy carbon electrode for electrochemical studies of hydroxychloroquine in the presence of acetaminophen. Colloids Surf. B Biointerfaces.

[45] Khalil M.M., El-aziz G.M.A., Ashry A. (2018). Potentiometric sensors based on hydroxychloroquine-phosphotungstate ion-pair and β-cyclodextrin ionophore for improved determination of hydroxychloroquine sulfate. J. Iran. Chem. Soc..

[46] George J.M., Mathew B. (2021). Cyclodextrin-mediated gold nanoparticles as multisensing probe for the selective detection of hydroxychloroquine drug. Korean J. Chem. Eng..

[47] Martínez-Huitle C.A. (2021). Environment-Friendly Electrochemical Processes. Materials.

[48] Ganiyu S.O., Martínez-Huitle C.A., Oturan M.A. (2021). Electrochemistry Electrochemical advanced oxidation processes for wastewater treatment: Advances in formation and detection of reactive species and mechanisms. Curr. Opin. Electrochem..

[49] Martínez-Huitle C.A., Panizza M. (2018). Electrochemical oxidation of organic pollutants for wastewater treatment. Curr. Opin. Electrochem..

