# Supplementary material for: Green Composite Sensor for Monitoring Hydroxychloroquine in Different Water Matrix
Source: Materials (Basel). 2021 Aug 31;14(17):4990. doi: 10.3390/ma14174990 (PMC8434169; doi:10.3390/ma14174990)
Supplement: Supplementary file 1 [file materials-14-04990-s001.zip › materials-1328370-supplementary.pdf]

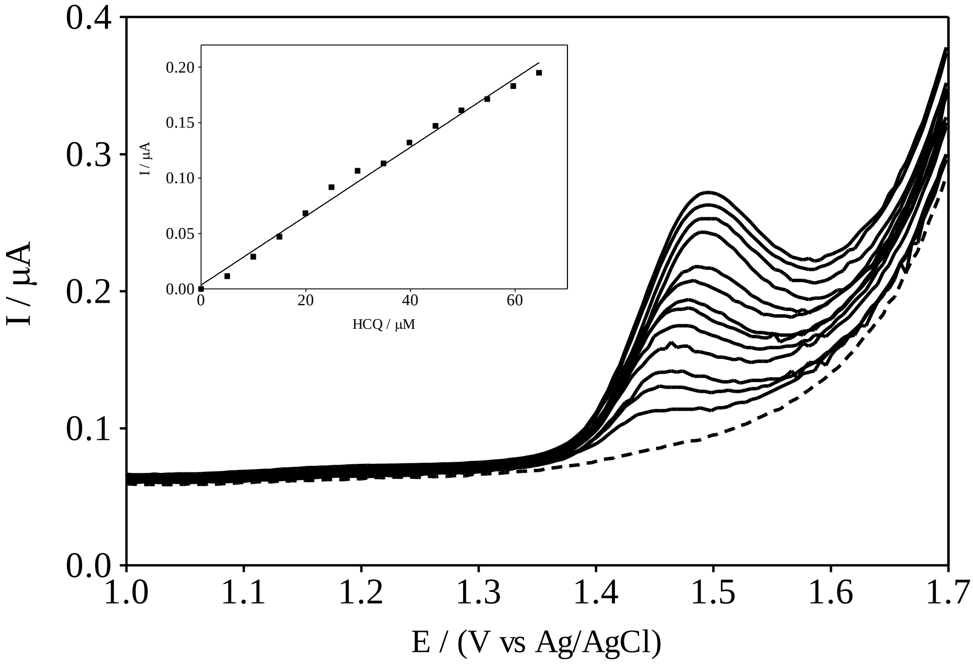

Figure S1. DPV curves for electrochemical cork-graphite sensor in acidified sample of lagoon by adding standard HCQ solution (10 mM) in well-known volumes to obtain: (a) 5,0 (b) 10, (c) 15, (d) 20, (e) 25, (f) 30, (g) 35, (k) 40, (l) 45, (m) 50, (n) 55, (l) 65, (k) 70 μM. Inset: Plot of electrochemical responses, in terms of current, as a function of HCQ concentration. Equation: Ipa (μA) = $3.13 \times 10^{-3} \times$ [HCQ] − 5.53×10^{-4} (R^2 = 0.993).
